# A jury of scientists: Formal education in biobehavioral sciences reduces the odds of punitive criminal sentencing

**DOI:** 10.1002/bsl.2588

**Published:** 2022-08-17

**Authors:** Mia A. Thomaidou, Colleen M. Berryessa

**Affiliations:** ^1^ Leiden University Faculty of Social and Behavioral Sciences Leiden Netherlands; ^2^ Rutgers University School of Criminal Justice Newark New Jersey USA

**Keywords:** education, free will, punishment, science, sentencing

## Abstract

This study examines how formal education in biological and behavioral sciences may impact punishment intuitions (views on criminal sentencing, free will, responsibility, and dangerousness) in cases involving neurobiological evidence. In a survey experiment, we compared intuitions between biobehavioral science and non‐science university graduates by presenting them with a baseline case without a neurobiological explanation for offending followed by one of two cases with a neurobiological explanation (described as either innate or acquired biological influences to offending). An ordinal logistic regression indicated that both science and non‐science graduates selected significantly more severe punishments for the baseline case as compared to when an innate neurobiological explanation for offending was provided. However, across all cases, science graduates selected significantly less severe sentences than non‐science graduates, and only science graduates' decisions were mediated by free will and responsibility attributions. Findings are discussed in relation to scientific understandings of behavior, the impact of science education on attitudes towards punishment, and potential criminal‐legal implications.

## 1 INTRODUCTION

Debates on the purposes of retributive versus consequentialist criminal punishments remain ongoing (Canton, 2018; Katz & Fondacaro, 2021). Yet, regardless of the purpose, both theoretically aim to punish an agent perceived to be morally responsible for the offense (von Hirsch, 2007). The U.S. Sentencing Commission acknowledges that sentencing has a range of philosophical goals that combine retribution, restoration, rehabilitation, and deterrence or incapacitation as the purposes of punishment (“U.S. Sentencing Commission,” 1995; Rappaport, 2003). Yet the notion of agency, or the seeming capability of a morally responsible agent to make willful, moment‐to‐moment choices, sits at the core of the normative concept of punishment; it is those choices that one arguably should be responsible for, as they are a product of free will, defined broadly as the continuous control one has over their direct actions (Reynolds & Placido, 2020) and the ability to *do otherwise* under the same conditions (Harris, 2012). Moral responsibility, tied to a supposed agent's freedom of will, is therefore considered a central element to criminal sentencing, and particularly as it relates to retribution (Duff, 1990). In this sense, punishment attributions are tied to the capacity for and judgments of free will (Daly, 2015).

At the same time, research in behavioral and biological sciences has begun to address questions about free will, as well as the control of and responsibility for a range of behaviors (Bargh & Chartrand, 1999; Daly, 2015; Gazzaniga, 2011). In recent years, neuroscience findings have begun informing our understanding of factors that influence different behaviors and how these may relate to legal criteria for responsibility, such as the ability for rational thought (Daly, 2015; Shariff et al., 2014). Ultimately, science may not suggest that humans behave without rationality, but rather that complex mental processes, sometimes outside of immediate cognitive control, can bear on decisions and behaviors that are not completely and freely willed (Katz & Fondacaro, 2021). Indeed, biobehavioral understandings of responsibility have been argued to be at odds with the assumption that humans always possess the faculty of moment‐to‐moment deliberate action and free will (Daly, 2015; Greene & Cohen, 2004; Harris, 2012).

Cashmore (2010) asserts that the criminal‐legal system operates “on the assumption that people make choices that do not simply reflect a summation of their genetic and environmental history” (p. 4499), with scientific research suggesting, however, that this assumption of absolute internal control is largely false. In the current study, we examine attitudes towards the punishment of offenders with proposed innate and acquired biological influences on their criminal behavior. As previous studies have shown that education in science could change people's attitudes about punishment (Katz & Fondacaro, 2021; Shariff et al., 2014), we particularly look to assess how a formal scientific education in biological or behavioral sciences may impact sentencing determinations.

### 1.1 Scientific understandings of behavior in the context of criminal justice

Research in the biological and behavioral sciences has continued to challenge the notion of free will (Bargh & Chartrand, 1999; Gazzaniga, 2011), as well as to uncover factors that may increase individuals' risk of committing antisocial or criminal acts (Barnes et al., 2015; Glenn & Raine, 2014). Human behavior has been shown to be influenced by factors that may be considered actively outside one's cognitive control (Bargh & Chartrand, 1999; Greene & Cohen, 2004). For example, research has shown significant influences on behavior as a result of factors such as mental illness (Rueve & Welton, 2008), physical and functional irregularities (Ling et al., 2019; Yang & Raine, 2009), genetically predisposed personality characteristics (Ferguson, 2010; Mednick & Kandel, 1988), or simply more general life experiences (Greene & Cohen, 2004). At the intersection of biobehavioral science and criminal justice, biosocial criminology addresses the fact that humans are sometimes at least partially “wired for behaviors” (Sokolowski & Corbin, 2012), with research gradually suggesting that free choices and individual responsibility may be inaccurate in describing many of the factors that can bear on later criminal behavior (Fondacaro & O’Toole, 2015; Greene & Cohen, 2004).

From this perspective, biological influences on criminal behavior can be broadly categorized as internal (innate neurobiological characteristics) and external (factors acquired through the environment and life experiences; Raine et al., 1997; Walt & Jason, 2017). A significant body of research suggests that innate predispositions may increase the likelihood of antisociality and offending. For instance, mutations of the monoamine oxidase A (MAO‐A) gene and other genetic influences have been linked to an increased risk of antisocial behavior (Ling et al., 2019; Moore et al., 2002). Studies have also found that many violent offenders commonly suffer from similar innate impairments in cognitive functioning (Raine et al., 1994, 2000). Importantly, recent advances in behavioral genetics indicate that innate traits may be expressed differently depending on a person's experiences (Ferguson, 2010). Indeed, it is thought that adverse environmental conditions can exacerbate innate predispositions toward psychopathology, whereas favorable conditions might compensate for them (Arseneault et al., 2000; Raine et al., 1997).

Acquired neurobiological characteristics have also been shown to be at least as important in influencing the development and exhibition of violence and criminal behavior (Raine et al., 1997; Walt & Jason, 2017). Reduced physiological reactivity, which may partly stem from acquired influences in early life, has been consistently found in antisocial individuals and links to a pattern of pathological under‐arousal (Fairchild et al., 2008; Ortiz & Raine, 2004). There also is a consistent body of research connecting prenatal nicotine and alcohol exposure with increased risk for criminality later in life (Brennan et al., 1999; Wakschlag et al., 2002). Acquired congenital physical anomalies, such as brain tumors or traumatic brain injuries, and environmental adversity have been associated with increased aggression, impulsivity, and violent offending (Arseneault et al., 2000; Kandel & Mednick, 2006). Thus, literature suggests that acquired factors may affect behavior through a variety of biobehavioral processes that take place outside one's own control (Carpenter et al., 2007; Coates, 2010; Fox et al., 2015; Grey, 2012; Neigh et al., 2009; Perry et al., 1995).

Behavior sometimes represents irrepressible outcomes as influenced by neurobiological systems (Raine & Yang, 2006). As such, neurobiological findings could be potentially useful during criminal sentencing by informing decision‐makers about potential behaviors that may be seriously and sometimes irrepressibly shaped by innate and environmental factors (Batastini et al., 2018; Mulvey & Iselin, 2008; Wayland, 2007). It has been argued that evidence or theories on the criminogenic effects of innate and acquired characteristics may help to align scientific findings with appropriate sentencing practices (Atiq & Miller, 2018; Connell, 2003; Wayland, 2007). Yet, an oversimplified, confusing, or unconvincing version of scientific knowledge is often what is ultimately considered in criminal justice settings (Farahany, 2016; Greene & Cohen, 2004).

### 1.2 Impact of biobehavioral science on punishment

Scholars have suggested that there are several scientific challenges against retribution, including empirical challenges to free will, that may raise theoretical questions regarding the moral justifications of punishment (Fondacaro & O’Toole, 2015; Katz & Fondacaro, 2021). Indeed, understanding human behavior as mechanistic and predetermined by factors beyond active control has been found to reduce lay support for retributive punishments (Shariff et al., 2014). At the same time, other research has found that adhering to retributive impulses in legal decision‐making may be costly, ineffective in reducing crime, and increasingly difficult to justify (Aizer & Doyle, 2015; Baćak et al., 2019; Fondacaro & O’Toole, 2015). Thus, an increasing number of scholars have attempted to shift the focus away from retributive, and instead towards restorative, rehabilitative, and less punitive forms of punishment (Braithwaite, 2002; Fondacaro & O’Toole, 2015; Gordon & Fondacaro, 2018; Greene & Cohen, 2004; Zedner, 1994). This deterministic approach has been widely adopted in recent literature, aiming to address more traditional punishment goals while also recognizing the scientific view that there may not always be a single agent morally responsible for each behavioral outcome (Carey & Paulhus, 2013; Gordon & Fondacaro, 2018; Koppel et al., 2018). Indeed, many scholars are increasingly suggesting that our evolving understanding of the neurobiology of will and behavior necessitates a move away from retribution and towards more consequentialist responses to crime (Carey & Paulhus, 2013; Katz & Fondacaro, 2021).

In legal systems with arguably and most often little expertise or focus on biobehavioral science, the distinction between innate and acquired influences on neurobiology and behavior may also be important. Existing experimental and other empirical literature has found that evidence of innate and acquired behavioral effects may result in differential sentencing determinations (Appelbaum & Scurich, 2014; Barnett et al., 2007; Berryessa, 2018a, 2018b; Davidson & Rosky, 2015). Some studies even show that innate genetic evidence, in some cases, may have a stronger mitigating effect on sentencing as compared to evidence on acquired neurobiological characteristics, such as brain damage from an accident and (Appelbaum & Scurich, 2014; Denno, 2011; Guillen Gonzalez et al., 2019).

For example, Appelbaum and Scurich (2014) raised concerns about the impact of genetics on perceptions of dangerousness, as they found that behavioral or neurobiological characteristics were seen as more mitigating in sentencing than genetic explanations for criminal behavior. Berryessa (2018b) found that judges linked their biases toward mental disorders with known genetic stigmas, leading to predilection for more severe warranted punishments. Moreover, innate characteristics can often be considered –based on popular belief– to be immutable and resistant to treatment (Berryessa, 2020). In criminal sentencing, a mental illness may be considered aggravating to sentencing when it is deemed unchangeable or when the offender is perceived as non‐compliant to treatment (Glannon, 2014; Xu et al., 2021). Yet, there is no concrete evidence in scientific literature that suggests that acquired criminogenic characteristics, such as trauma or brain tumors, actually allow individuals more freedom of will than people that are innately predisposed to offending behavior.

Thus, as science has begun unravelling the web of influences on behavior, stemming from biopsychosocial elements that can be conceptualized as factors outside one's immediate control (Katz & Fondacaro, 2021), advances in our understanding of the dual impact of biology and experiences on behavior may be increasingly important to consider when considering punishment (Allen et al., 2019; Greene & Cohen, 2004; Shariff et al., 2014). Based on this literature, a person's failure to exhibit a desirable or expected behavior may not reasonably fully be explained in terms of the choices of a free agent, but must also be considered in the context of underlying biological characteristics which bear on behavior due to both internal and external factors (Gordon & Fondacaro, 2018). Indeed, Ling et al. (2019) argue that the evidence of abnormal neurobiological characteristics in people with violent and criminal tendencies may lead us to increasingly recognize a lack of will and affected moral responsibility in offenders with such characteristics.

It is important to know that, while evidence of an inability to freely choose one's behavior could minimize justifications for retributive punishment (Shariff et al., 2014), challenging the notions of free will and moral responsibility does not necessitate that an individual cannot face consequences for their behavior (Gordon & Fondacaro, 2018). Biobehavioral science recognizes that offenders could be punished for a criminal act in order to protect society, or as a strategy to deter them from future harm through rehabilitation. But if one believes that an offender engaged in a criminal behavior as influenced by factors that, at least in part, may have compromised their free ability to do otherwise, it is the motivation for punishment that may change (Katz & Fondacaro, 2021). Thus, scientific understandings of behavior may open a path to a more holistic understanding of –and response to– factors that influence criminal behavior (Gilligan, 1997).

### 1.3 Scientific knowledge informs attitudes towards criminal justice

Science has also been argued to potentially inform and change people's attitudes about and support for punishment (Katz & Fondacaro, 2021; Shariff et al., 2014). Anti‐free‐will perspectives resonate beyond science and academia, with the media and popular opinion pointing to increasingly mechanical causes for behavior and questions about the mental and moral consequences of reduced free‐will (Baer et al., 2008; Greene & Cohen, 2004; Nahmias, 2011; Shariff et al., 2014). Therefore, evolving knowledge on free will and behavior may potentially update societal notions on individual responsibility and support for particular modes of sentencing (Burrows & Reid, 2011; Roberts & de Keijser, 2014).

Such a shift in society's views on offending behavior may also be important in the context of the democratization of punishment (Gebotys & Roberts, 1989; Roberts & de Keijser, 2014). Roberts and de Keijser (2014) discuss that sentencing councils and commissions often focus on popular views and routinely consider public opinion research and community perspectives in amending sentencing guidelines. Indeed, researchers have argued that shifting public intuitions about punishment should be taken into consideration by sentencing authorities and policy‐makers (Xu et al., 2021). In a legitimate and fair criminal justice system, existing punishment practices may need to be better tailored to align with public views (Robinson, 2013).

Yet shifts in public attitudes about sentencing may not necessarily be uniform across society. Shariff et al. (2014) argue that exposure to knowledge or education in biobehavioral science and research on biological influences on behavior may alter the way that some people understand free will and attribute responsibility, which may lead individuals to have differing views on and support for punishment practices. Scientific information makes its way into many people's lives through the media, with only a section of society being formally educated in biological and behavioral sciences (Hanson, 2021; Merlino et al., 2002).

Receiving formal education in an area has been considered as a form of conceptual expertise (Kahneman, 2011), as higher education appears to involve an understanding of intricate relationships among factors and context‐sensitive application of such knowledge (Kahneman, 2011; Kahneman & Klein, 2009; Shafto & Coley, 2003). Indeed, results in the literature show that graduates and professionals in a given field can be distinguished based on specific aspects of their expertise (Asamizuya et al., 2022; Kahneman, 2011; Savadori et al., 2004; Slovic et al., 1979; Smith et al., 2013). For example, there are differentiations in the thinking patterns of biology and non‐biology college majors (Smith et al., 2013), as well as in the cognitive strategies and solutions employed by experienced fishermen as compared to novices (Shafto & Coley, 2003) or legal experts versus lay persons (Asamizuya et al., 2022). Thus, education in biological and behavioral sciences may be considered broadly as a type of conceptual expertise on behavior, which may lead science graduates to think differently about free will, individual responsibility, and ultimately, about modes and support for criminal punishment in certain contexts (Busby‐Mott, 1992; Kahan, 2010; Slovic & Västfjäll, 2010; Xu et al., 2021).

In many cases, the potential influence of scientific knowledge and education on sentencing could be indirect. Many studies have addressed what underlies support for and determinations of punishment (e.g., Aspinwall et al., 2012; Berryessa, 2018a; Koppel et al., 2018; Mulvey & Iselin, 2008; Shariff et al., 2014), generally suggesting that factors such as moral responsibility, free will, and the dangerousness of an offender may compete and be differentially assessed when considering preferences and support for sentencing (Aspinwall et al., 2012; Berryessa, 2018a; Mulvey & Iselin, 2008; Nahmias et al., 2008). Indeed, as they relate to the freedom of control over one's behavior and the societal impact of criminality (“U.S. Sentencing Commission,” 1995; Rappaport, 2003), it is unsurprising that differential considerations of these factors might very well influence support for and considerations in sentencing (Shariff et al., 2014). Thus, if formal scientific education is able to shape individuals' understanding of biology and behavior (Kahneman, 2011; Richards, 2015), it is possible that the growing scientific evidence of innate and acquired influences on behavior could impact people's views on behavioral control and dangerousness, which may correspondingly affect their views toward sentencing.

To our knowledge, no empirical research has been yet conducted to examine the punishment perspectives of people with formal science education as compared to those without such education, including in more intricate contexts involving the consideration of types of neurobiological explanations for offending. Investigating evolving public views on sentencing remains an important component of legitimacy within a criminal justice that is increasingly confronted with scientific knowledge (Bottoms & Tankebe, 2012; Jackson et al., 2017; Roberts & de Keijser, 2014). Indeed, as neurobiological evidence appears to become more increasingly involved in sentencing (Farahany, 2016), such an examination may help us not only understand how scientific information may be understood and considered by those with a scientific background, but also what changes in punishment perspectives may lay ahead, in an increasingly scientifically‐informed society (Gebotys & Roberts, 1989; Roberts & de Keijser, 2014).

### 1.4 Current study

The current research, using a sample of university graduates with majors in both science and non‐science fields, examines how formal education in biological and behavioral sciences may impact attitudes toward punishment. We assessed their views on free will, moral responsibility, dangerousness, and criminal sentencing in situations involving neurobiological explanations of behavior that have been used in previous paradigms (Berryessa et al., 2021). We aim to build on existing literature on how people judge offenders with different biobehavioral characteristics to specifically examine how science education may affect support for sentencing in these contexts, as well as discuss the potential implications of science becoming more widespread and popularized with regard to the criminal‐legal system.

Our primary research question was whether, when compared to non‐science graduates, individuals with formal education in biological and behavioral scientific fields would favor less punitive and potentially more treatment‐focused punishment approaches for offenders described as having neurobiological influences on their behavior. Based on literature examining attitudes towards innate and acquired neurobiological influences on behavior (Appelbaum & Scurich, 2014; Barnett et al., 2007; Berryessa, 2018a, 2018b; Davidson & Rosky, 2015), we also employed a manipulation to examine whether presenting offenders as having either innate or acquired biobehavioral characteristics would impact sentencing determinations. Finally, we were also interested in whether differential punishments between science and non‐science graduates would be mediated by their perceptions of free will, responsibility, and/or dangerousness.

We hypothesized that people with a biobehavioral science education would be more likely to support less punitive sentencing options, as compared to non‐science graduates, in instances in which the offender's behavior was said to be either acquired or innately neurobiologically influenced. Indeed, previous studies show that shifts in people's free‐will beliefs, even by simply learning about brain function, can impact attitudes regarding moral responsibility and criminal sentencing (Shariff et al., 2014). We expected such mitigating effects to be differentially mediated between science and non‐science graduates by beliefs about free will (as these are shown to impact perceptions of responsibility and sentencing determinations; Daly, 2015; Shariff et al., 2014), moral responsibility (as attitudes about responsibility are key in sentencing determinations; Carlsmith et al., 2002), and views on dangerousness (as dangerousness is known to influence support for deterrence and incapacitation as punishment objectives, but has been less tied to individual responsibility; Allen et al., 2019; Aspinwall et al., 2012; Davidson & Rosky, 2015).

## 2 METHODS

### 2.1 Participants

The targeted sample was international university graduates (18 years or older) able to fill in an online survey and with a good command and understanding of the English language. Participants were recruited internationally through university networks via email and posts on social media such as Twitter and Linkedin. We used a snowball sampling procedure, in which participants were asked to refer other university graduates as potential participants (Atkinson & Flint, 2001; Zhong et al., 2014). Initial recruitment targeted graduates from the researchers' network at European and U.S. universities. Snowball sampling methods are often used for their logistic convenience in order to gain access specific or inaccessible populations, as well as methodologic advantages (Atkinson & Flint, 2001; Zhong et al., 2014) such as obtaining a diverse international sample for this study.

An a‐priori sample size calculation was performed using G*Power version 3.1.9.4, based on a similar study with comparable outcome measures and analyses (Berryessa, 2021). The required effect size was based upon four outcome measures. The primary research question was between‐groups, while secondary ones included within groups measures. Based on the power analysis, a sample of 160 participants was targeted, which is enough for sufficient power for *f* = 0.25, power = 0.80, *df* = 2, for 2 different groups in the primary analysis. Ethical approval was obtained by the author's institutional ethics committee (2021‐07‐16‐V2‐xxxx) and the study was pre‐registered on ClinicalTrials.gov (NCTxxxxxx14).

### 2.2 Design and procedure

**FIGURE 1:**
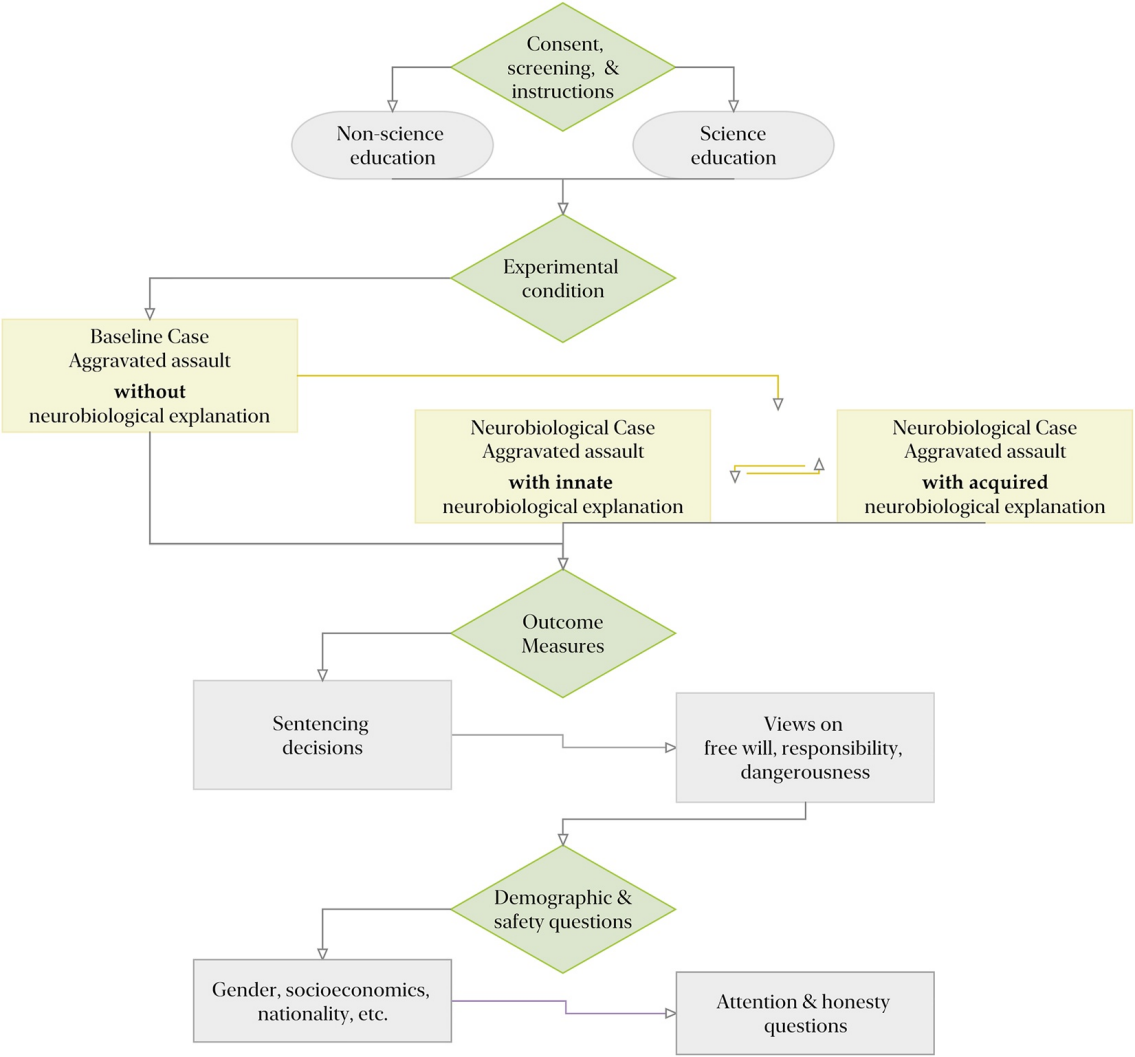
The survey design. All participants read the baseline case, following which half of the participants were exposed to a case with an innate neurobiological defense and the other half read the case of an offender with an acquired neurobiological disorder

**FIGURE A1:**
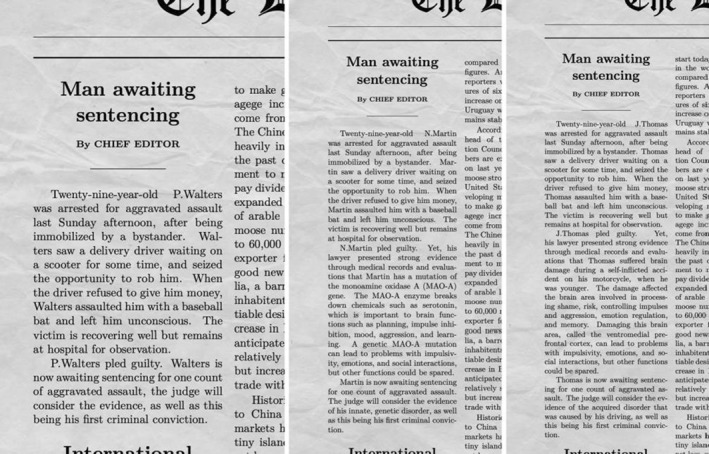
The three presented fictional criminal cases

The current research employed a within‐between, experimental design via Qualtrics, that took approximately 15 min to complete. As outlined in Figure 1, after providing consent for participation and answering several screening questions about study eligibility, each participant saw two out of three different vignettes that described similar criminal cases. In each criminal case, the same aggravated assault incident was described through the format of a newspaper article, featuring young male first‐time offenders that had been found guilty of inflicting severe bodily injury in an attempt to rob a delivery driver. This was based on vignettes published previously in related studies (Berryessa, 2017b; Berryessa et al., 2021). Each case was the length of two short paragraphs and did not contain extensive details (see also Appendix A: Figure A1). Before the presentation of the vignettes, a short definition and description of the criminal charge of aggravated assault was provided.

All participants were first presented with the *baseline* case, in which no information was given regarding the offender's defense and answered a set of questions. Participants were then randomly assigned one of two near identical cases, that now additionally presented either evidence of the offender's *innate* (a genetic mutation; Caspi et al., 2002) or *acquired* (a head injury; Moore et al., 2019) neurobiological disorders, and then answered the same set of questions about this second case. The main independent variable was thus the type of *case* presented. The only difference between the three cases, and the main manipulation studied here, was information provided about the offender's background and the type of neurobiological evidence presented:

1. *Baseline case* presented to all participants with no mention of a neurobiological defense: “P. Walters pled guilty. Walters is now awaiting sentencing for one count of aggravated assault, the judge will consider the evidence, as well as this being his first criminal conviction.”
2. *Innate case* presented to half of the participants: “N. Martin pled guilty. Yet, his lawyer presented strong evidence through medical records and evaluations that Martin has a mutation of the MAO‐A gene. The MAO‐A enzyme breaks down chemicals such as serotonin, which is important to brain functions such as planning, impulse inhibition, mood, aggression, and learning. A genetic MAO‐A mutation can lead to problems with impulsivity, emotions, and social interactions, but other functions could be spared. Martin is now awaiting sentencing for one count of aggravated assault. The judge will consider the evidence of his innate, genetic disorder, as well as this being his first criminal conviction.”
3. *Acquired case* presented to half of the participants: “J. Thomas pled guilty. Yet, his lawyer presented strong evidence through medical records and evaluations that Thomas suffered brain damage during a self‐inflicted accident on his motorcycle, when he was younger. The damage affected the brain area involved in processing shame, risk, controlling impulses and aggression, emotion regulation, and memory. Damaging this brain area, called the ventromedial prefrontal cortex, can lead to problems with impulsivity, emotions, and social interactions, but other functions could be spared. Thomas is now awaiting sentencing for one count of aggravated assault. The judge will consider the evidence of the acquired disorder that was caused by his driving incident, as well as this being his first criminal conviction.”

### 2.3 Measures

Following each of the two vignettes that were presented to them, participants answered a set of questions. We first asked participants to select one of five *sentences* for each offender, ranging from least severe to most severe: (a) *treatment* (no punishment); (b) a *non‐custodial* punishment; (c) *maximum 1‐year* imprisonment; (d) *1–5 years* imprisonment; or (e) a *5‐year minimum* imprisonment.

Participants were also asked to rate (a) how much *free will* they perceived each offender to have [from 0 (no freedom of will) to 100 (complete freedom of will), how much free will do you think the offender has in his behavior?], (b) how much *moral responsibility* they perceived each offender to have [from 0 (not at all responsible) to 100 (completely responsible), how much responsibility do you think the offender has for his behavior?], as well as (c) how *dangerous* each offender was assumed to be [from 0 (not at all dangerous) to 100 (completely dangerous), how dangerous do you think the offender is?]. After completing their responses for both criminal cases, participants were asked to rate how much free will they think that humans have in *general*. All answers were provided on a scale from 0 to 100. These measures were adapted from previous experimental vignettes studies examining punishment intuitions (Berryessa, 2017a, 2020; Katz & Fondacaro, 2021; Shariff et al., 2014). No definitions were specified for these outcome measures, so to allow participants to respond based on their own understanding of such concepts.

As an attention check, we asked participants to select a logical numerical response that confirmed whether they were paying attention and carefully reading the survey questions. We also asked participants if they provided honest responses during the study. Basic demographic variables were also collected and used in analyses as controls, included the lived gender, age, criminal history, occupation, income, and questions regarding participants' education (see Table 1).

**TABLE 1:** Descriptive statistics. Descriptive statistics and sentences selected, shown as percentages of the total sample, as well as cross‐tabulated between people with and without a science education

	Percent of total sample (*n* = 160)	Percent of total *science education* (*n* = 77)	Percent of total *non‐science education* (*n* = 83)
Lived gender			
Woman	83%	48.2%	51.8%
Man	17%	48.1%	51.9%
Occupation			
Employed full/part‐time	68.2%	46.8%	53.2%
Masters/PhD student	21.2%	66.3%	33.7%
Raising children or retired	10.6%	32.2%	67.8%
Income			
Below 500 p/m	12.4%	50%	50%
500–1200 p/m	9.4%	40%	60%
1200–2000 p/m	13.7%	31.8%	68.2%
2000–3000 p/m	31.9%	56.9%	43.1%
Above 3000 p/m	32.5%	51.9%	48.1%
Criminal history			
No encounters with the law	53.1%	43.5%	56.5%
Traffic violation	40%	56.3%	43.7%
Arrest but no conviction	0.6%	0%	100%
Close family member incarcerated	5%	50%	50%
Convicted or incarcerated	1.4%	0%	100%
Sentence *baseline* case			
Treatment	9.4%	80%	20%
Non‐custodial	10.6%	70.6%	29.4%
Max 1 year	22.5%	47.2%	52.8%
1–5 years	49.4%	39.2%	60.8%
5‐year min.	8.1%	38.5%	61.5%
Sentence *innate* case			
Treatment	41.7%	57.1%	42.9%
Non‐custodial	7.1%	83.3%	16.7%
Max 1 year	14.3%	33.3%	66.7%
1–5 years	22.6%	31.6%	68.4%
5‐year min.	4.8%	25%	75%
Sentence *acquired* case			
Treatment	45.2%	47.4%	52.6%
Non‐custodial	13.1%	45.5%	54.6%
Max 1 year	17.9%	46.7%	53.3%
1–5 years	21.4%	33.3%	66.7%
5‐year min.	2.4%	0%	100%

*Note*: For the gender variable, we asked participants if they live as a man or as a woman.

**FIGURE 2:**
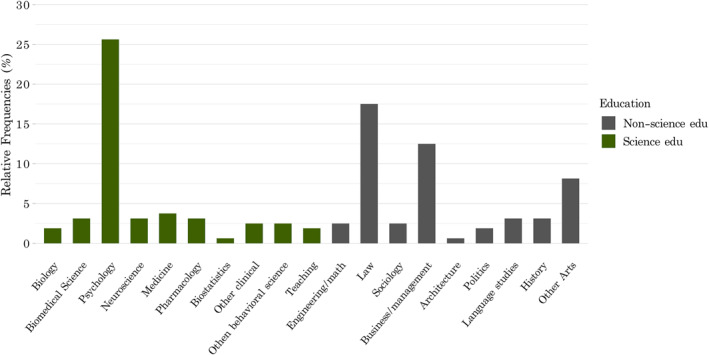
The different fields of study reported in the sample of university graduates, split between people with *science education* and *non‐science education*. While many fields of study may overlap, an effort was made to distinguish between individuals that have completed formal education in subjects related to biology and behavior, and those that graduated in unrelated fields

To examine the impact of scientific education as an individual‐level predictor, we categorized participants based on whether or not they were formally educated in biobehavioral sciences (such as medicine, neuroscience, or psychology). We asked participants to report the highest level of education that they have completed and hold a degree in. Figure 2 lists participants' fields of education and shows their division into biobehavioral science versus non‐science graduates. Being categorized as having received a biobehavioral *science education* does not suggest that participants in this group are specialized biobehavioral scientists, but rather that they have completed higher education is a scientific discipline related to human biology and/or behavior. The label *non‐science education* describes participants who did not receive a higher education in a biobehavioral science but have formal education in the arts, social sciences, or mathematics (see also Figure 2). While this partitioning is not perfect, an attempt was made to differentiate between people who have increased knowledge of human biology or behavior, as compared to graduates with no such formal education.

### 2.4 Data & analyses plan

For analysis, we used R programming software (R Core Team, 2022), MASS (Venables & Ripley, 2002), lavaan (Rosseel, 2012), stargazer (Hlavac, 2018), and ggplot2 (Wickham, 2016). First, we performed descriptive analyses and plotted the main variables of interest. There were no outliers present in the data (indicated by Mahalanobis distance), the assumptions of linearity and homogeneity were met, and the data appeared normally distributed. When checking among the independent variables for additivity, we found that *free will*, *responsibility*, and *dangerousness* were moderately correlated around *r* = 0.6, therefore we ran a main model with only *free will* as the main variable of interest, to control for multicollinearity, and also built the same model with all three variables included, to explore their influence on the model.

**FIGURE B1:**
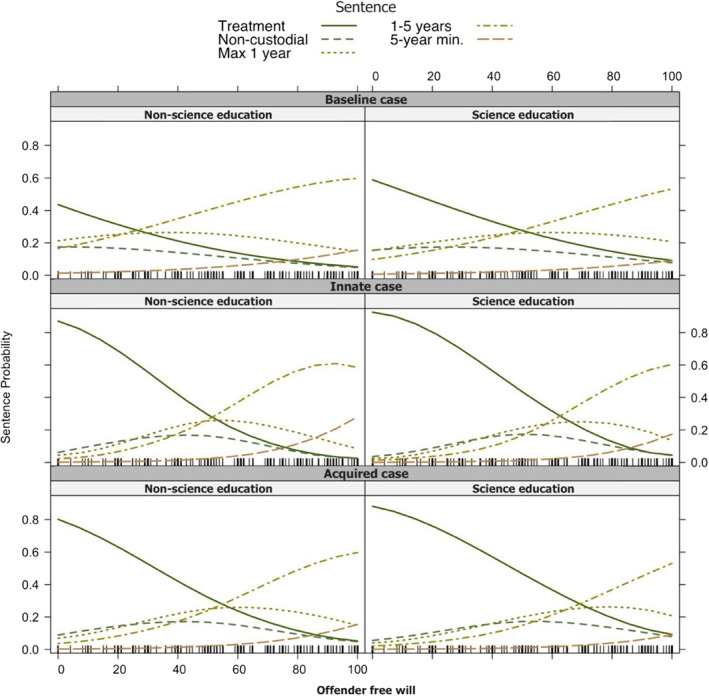
Sentencing probabilities by education, including the significant predictor of offender free will, based on the ordinal logistic regression model. The sharper slope changes in the neurobiological cases show that the supposed free will of the offender had a more decisive effect when the offender suffered from a neurobiological disorder. It is apparent that for the baseline case, the levels of offender free will did not affect sentencing decisions as strongly as for the neurobiological cases.

The main analysis was an ordinal logistic regression with the multi‐categorical outcome of *sentence*. The variables of interest were *case* (i.e., *baseline*, *innate*, or *acquired*), *education* (i.e., *science* vs. *non‐science education*), and their interactions. Additional variables in the model were *criminal history*, *age*, *income*, *offender free will,* and *general free will*. This analysis was performed using the Proportional Odds Logistic Regression (polr) function (Agresti, 2002; Venables & Ripley, 2002) on a randomly pulled training dataset (80% of total). Machine learning validations were performed on the (remaining 20%) testing set (Appendix B: Table B1 and Figure B1).

Based on the results of the logistic regression, we next conducted mediation analyses using Structural Equation Modeling, for the outcome of *sentencing*. We ran three mediation analyses with either *free will*, *responsibility*, or *dangerousness* as the mediator. The independent variable was *case* (*baseline*, *innate*, or *acquired*). We additionally ran each of these three models separately for participants with a *non‐science* versus biobehavioral *science education*. These path models were fitted using the Fit Structural Equation Models (sem) function (Rosseel, 2012) with a diagonally weighted least squares estimator. We estimated confidence intervals using bootstrapping at 1000 draws.

## 3 RESULTS

### 3.1 Participant characteristics

In total, 176 people participated in this study. 15 participants were excluded for not having completed higher education and one participant was excluded for failing the honesty question. This resulted in 160 included responses, split between people with a *science education* (*n* = 77) and *non‐science* graduates (*n* = 83). Figure 2 lists the fields of education and their allocation in the two groups. The mean age was *M* = 40 years (*SD* = 12.04; science education *M* = 39.3, non‐science *M* = 41.2). Table 1 lists percentages for demographics and Appendix C: Table C1 lists the countries of residence, nationalities, and criminal histories in each group.

### 3.2 Main results

**FIGURE 3:**
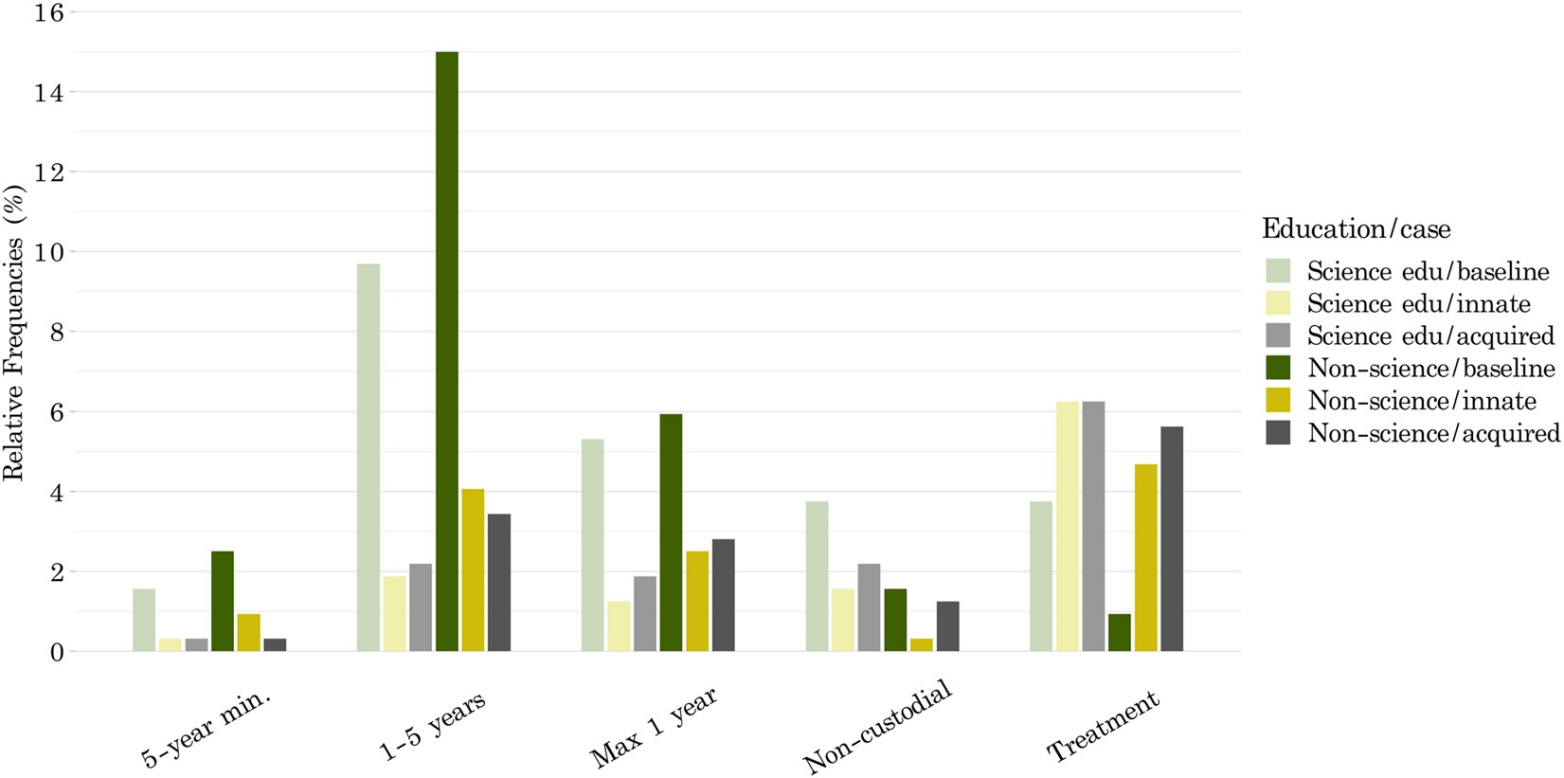
Sentencing decisions of graduates with a *science education* and graduates with *non‐science education* for the *baseline* case (green) versus *innate* (yellow) and *acquired* (grey). Science graduates are presented in faint colors. Biobehavioral science graduates tended to select less punitive sentences overall, compared to non‐scientists. Both groups seem to select more severe punishments for the baseline, compared to cases with a neurobiological defense

As seen descriptively in Table 1, *non‐science* graduates' sentences were generally concentrated in the middle to most severe options given for sentencing support, whereas people with a *science education* generally selected less severe options for sentencing overall (Figure 3).

**FIGURE 4:**
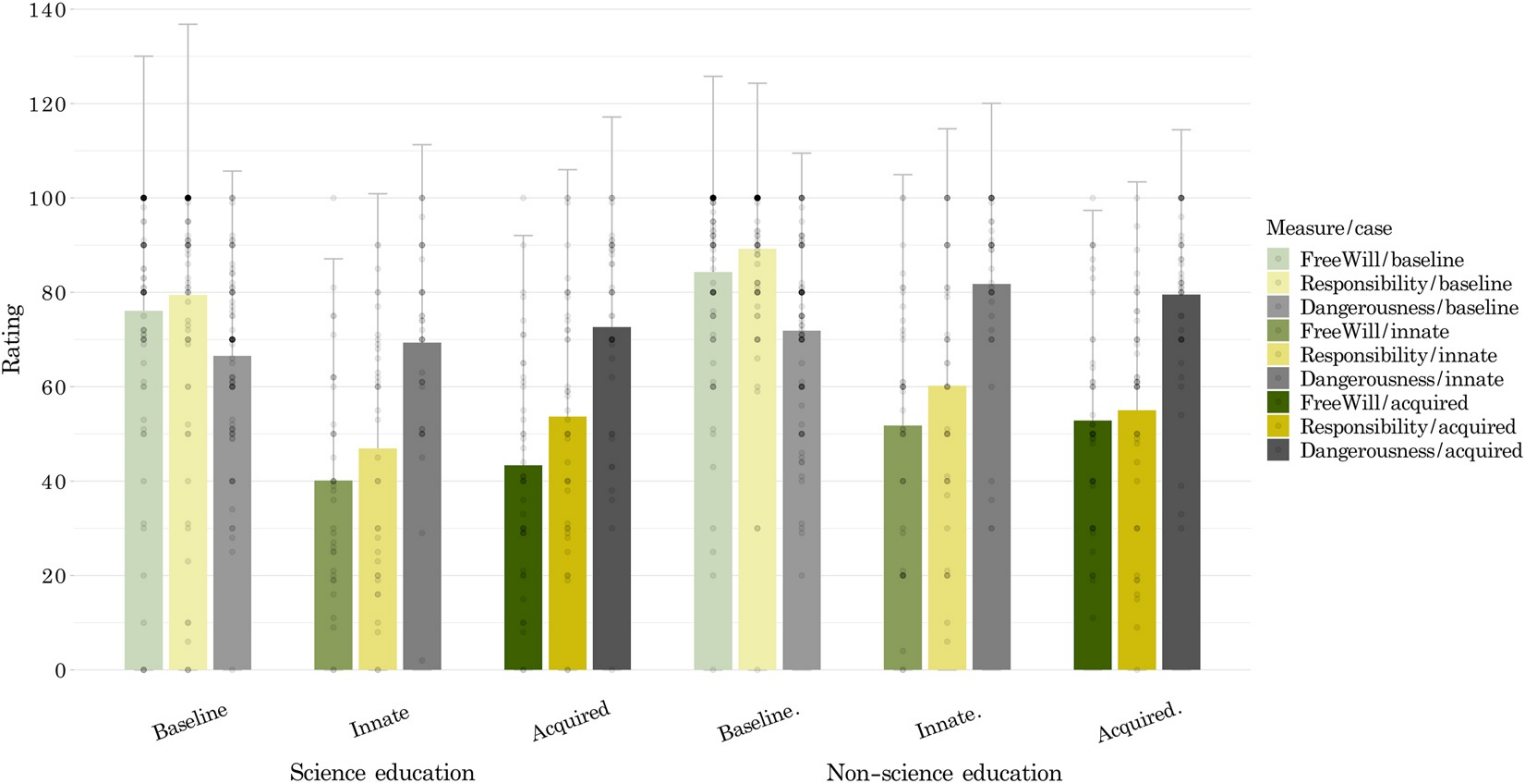
Mean ratings of the perceived *responsibility*, *free will*, and *dangerousness* of the offenders. The baseline case is presented in faint colors. People with a *science education* (left six columns) on average perceived the offender as being less responsible, less dangerous, and having lower freedom of will. Both groups rated cases with a neurobiological explanation as less responsible and free –but more dangerous– than the baseline case

**FIGURE 5:**
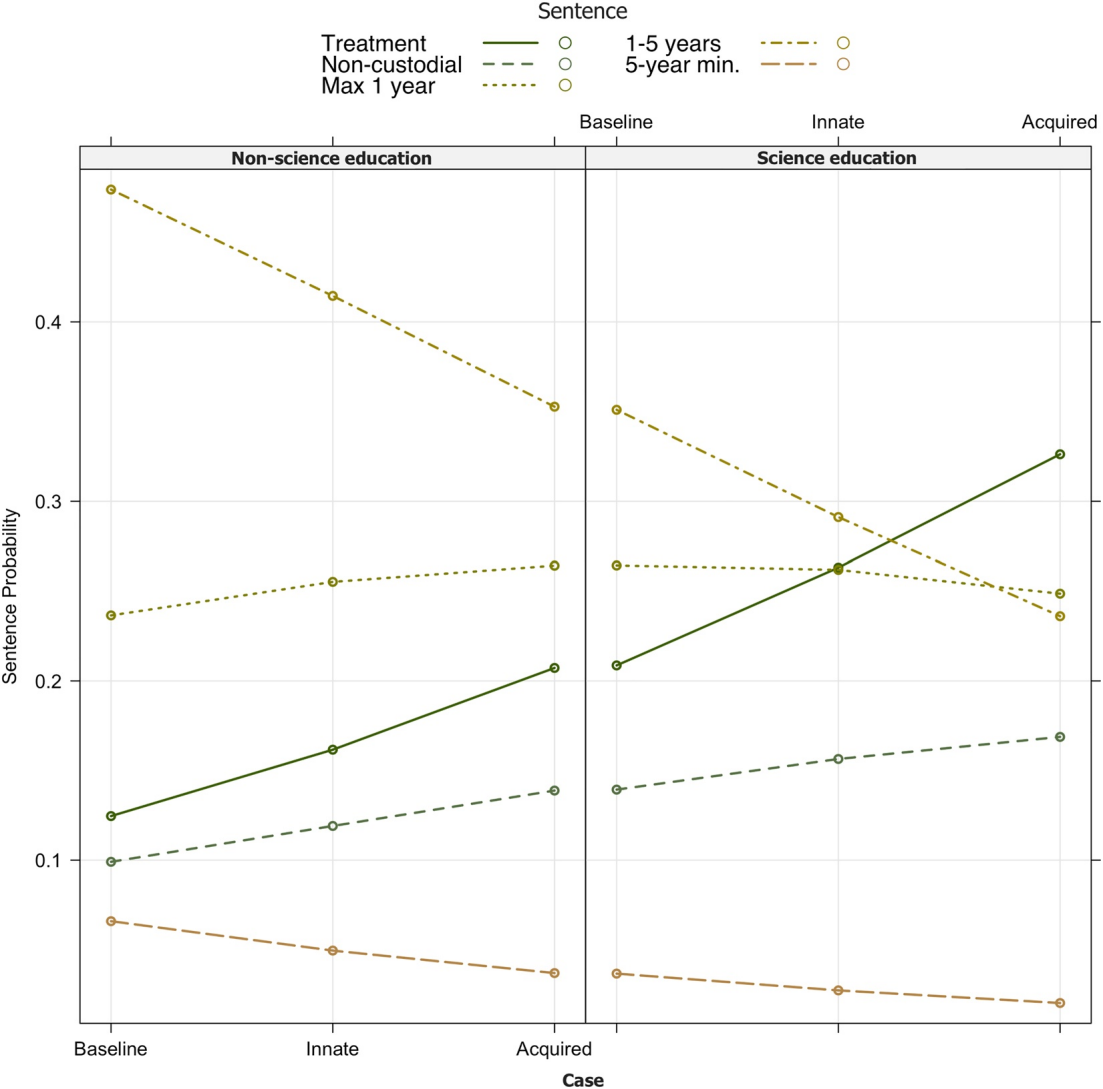
Sentencing probabilities for graduates with a *non‐science* versus biobehavioral *science education*. Those with a background in biobehavioral sciences selected significantly less severe sentences overall

Results of the ordinal log regression model showed that participants selected significantly more severe sentences for the *baseline* case as compared to an offender with an *innate* disorder (*b* = −2.46, *SE* = 0.92, *t* = −2.67, *p* = 0.007). For the *baseline* offender, the log odds of receiving a tougher *sentence* were 2.84 times those of the offender with an *innate* disorder. The difference between the *acquired* and *baseline* case did not reach significance (*p* = 0.14; Table 2). The analysis further indicated that, as seen in Figures 4 and 5, having a *science education* had a significant effect on *sentencing* (*b* = −0.70, *SE* = 0.26, *t* = −2.70, *p* = 0.006) as compared to *no science education*, with *science* graduates selecting significantly less severe sentences across all cases (Figure 5). When looking at the interaction of *education group* and the type of *case*, no significant differences were detected between people with a *science* versus *non‐science education* in the *sentences* given between the different types of *cases* (Table 2).

**TABLE 2:** Ordinal logistic regression. Ordinal logistic regression model of participants' sentencing decisions for all three criminal cases, as a function of independent, control, and demographic variables, as well as interaction terms

	*b*	Std. Error	*t*	Sig.
Education				
Science education	−0.70	0.26	−2.70	**0.006**
Age	0.002	0.01	0.24	0.81
Income				
500–1200 p/m	−0.18	0.55	−0.32	0.75
1200–2000 p/m	0.12	0.54	0.21	0.83
2000–3000 p/m	−0.05	0.41	−0.13	0.89
Above 3000 p/m	−0.26	0.43	−0.59	0.55
Offender free will	0.06	0.01	4.61	**<0.001**
General free will	−0.004	0.01	−0.50	0.61
Type of case				
Innate case	−2.46	0.92	−2.67	**0.007**
Acquired case	−1.31	0.89	−1.48	0.14
Case by education interactions				
Innate case *x* Science education	−0.34	0.62	−0.55	0.58
Acquired case *x* Science education	0.50	0.58	0.86	0.38
Case by free will interactions				
Innate case *x* free will	0.04	0.01	2.70	**0.006**
Acquired case *x* free will	0.01	0.01	0.60	0.55
Intercepts				
Treatment | non‐custodial	1.89	0.91	2.08	0.04
Non‐custodial | max 1 year	2.38	0.92	2.59	0.01
Max 1 year | 1–5 years	3.58	0.93	3.84	<0.001
1–5 years | 5‐year min.	6.41	0.97	6.59	<0.001

*Note*: For the *group* variable, *non‐science education* is the reference category and thus results under *Education* represent a comparison of *science* to *non‐science education*. For *income*, *below 500 p/m* is the reference category. For the type of *case* as well as for the interactions, the *baseline* case was the reference category. The residual deviance of this model was 632.5, while the Akaike Information Criterion (AIC) was 666.5. Significant effects for the coefficients are shown in bold.

The offender's supposed *free will* yielded a significant effect on *sentencing*. The results indicate that participants who assumed the offender had more *free will* selected significantly more severe *sentences* (*b* = 0.06, *SE* = 0.01, *t* = 4.61, *p* < 0.001). For each unit increase in perceived offender *free will* (rated on a 0–100 scale), we expect a 0.06 increase (6%) in the log odds of granting a more severe *sentence*, given that all of the other variables are held constant (Table 2). We also examined the interaction between offender *free will* and the type of *case* on sentencing (Figure 5). The results indicate that perceived *free will* significantly affected the sentencing disparity between the *baseline* and the *innate* case (*b* = 0.04, *SE* = 0.01, *t* = 2.70, *p* = 0.006), meaning the higher the perceived *free will*, the smaller the difference in sentencing between the two *cases* (i.e., the smaller the effect of having an *innate* disorder as an explanation for the criminal behavior). Lastly, measures of *general free will*, *age* and *income* did not have a significant effect on the sentencing (all *p* > 0.01, Table 2). Model performance validations and computed multiclass confusion matrices can be found in Appendix B: Table B1 and Figure B1.

### 3.3 Mediation results

**FIGURE 6:**
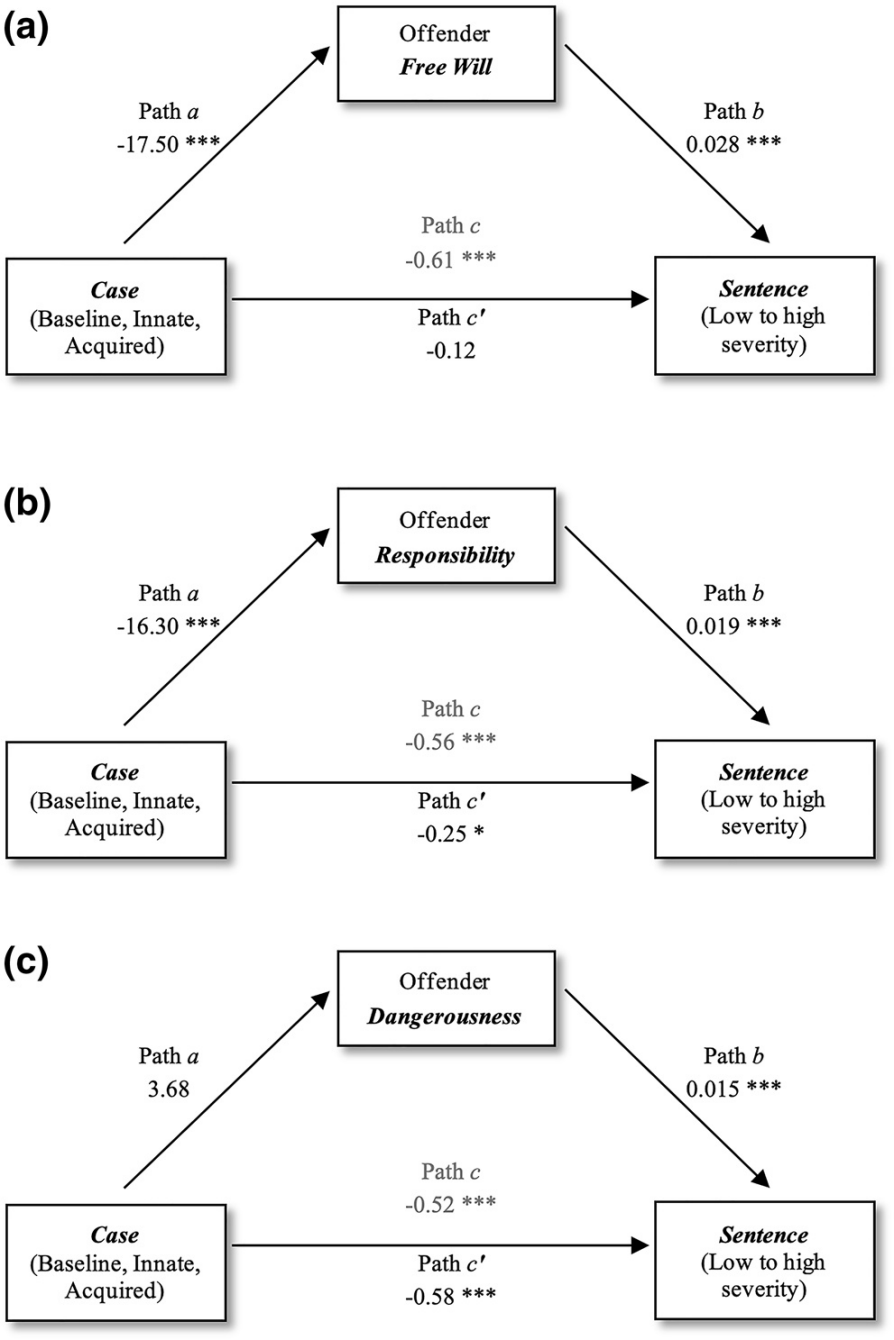
Diagram of the three main mediation models and results for computed paths. As expected, with the mediator of *free will*, all paths marked significant relationships, except for path c’ which showed that the relationship between *case* and *sentence* was no longer significant after controlling for perceived offender *free will* (a). When *responsibility* was treated as the mediator, there was only a partial mediation of the relationship between the type of *case* and the *sentence* severity (b). *Dangerousness* did not mediate the relationship between the type of *case* and the *sentence* severity (c). Values marked with three asterisks are significant at *p* < 0.001 while values marked with one asterisk are significant at *p* < 0.01

A diagram of the mediated relationships can be seen in Figure 6. All computed paths are shown in Appendix D: Table D1. **Path c** presents the total effect of *case* on *sentencing* for all cases (*b* = −0.61, *SE* = 0.09, *p* < 0.001, 95% CI [−0.76, −0.41], *R* ^*2*^ = 0.43) and indicates that the type of defense significantly predicted the severity of *sentences* selected. **Path a** (*b* = −17.50, *SE* = 1.65, *p* < 0.001, 95% CI [−20.72, −14.32]) shows that the offender's *free will* was rated as significantly higher in the case without a neurobiological explanation, also illustrated in Figures 3 and 6a. **Path b** (*b* = 0.028, *SE* = 0.004, *p* < 0.001, 95% CI [0.02, 0.03]) indicates that higher assumed *free will* led to more severe *sentences*. The indirect effect coefficient of *case* on *sentencing* through assumed offender *free will* was significant (path a*b *b* = −0.49, *SE* = 0.09, *p* < 0.001, 95% CI [−0.57, −0.24]). Full mediation was found, with the relationship between *case* and *sentence* no longer being significant after controlling for *free will* (**path c’** *b* = −0.12, *SE* = 0.09, *p* = 0.21, 95% CI [−0.37, 0.007]).

To examine whether this mediated effect differed between people with a *non‐science* versus *science education*, we ran the same path analysis separately for each education group. For those with a *science education*, we found again a significant mediation (path a*b *b* = −0.42, *SE* = 0.11, *p* < 0.001, 95% CI [−0.67, −0.26]). The relationship between *case* and *sentence* was no longer significant after controlling for *free will* (**path c’** *b* = −0.12, *SE* = 0.14, *p* = 0.41, 95% CI [−0.39, 0.16]). For people with a *non‐science education*, the indirect effect of *case* on *sentence* through *free will* was not significant (path a*b *b* = −0.43, *SE* = 0.26, *p* = 0.10, 95% CI [−0.57, 0.11]). The two separate mediation models show that people with a biobehavioral *science education* made sentencing decisions based on differential *free will* judgements of between the three *cases*, whereas *non‐science* graduates made *sentencing* decisions based on the type of *case,* but decisions were not mediated by *free will*.

We ran the same models with *responsibility* as the mediator variable (Figure 6b) and found a significant partial mediation (path a*b *b* = −0.31, *SE* = 0.05, *p* < 0.001, 95% CI [−0.42, −0.22]). The relationship between *case* and *sentence* showed a small drop in significance after controlling for *responsibility* (**path c’** *b* = −0.25, *SE* = 0.09, *p* = 0.004, 95% CI [−0.42, −0.08]). When examining this mediation separately for people with *science* versus *non‐science education*, we found that for *science* graduates, *responsibility* was a stronger mediator (total effect in **path c** *b* = −0.52, *SE* = 0.12, *p* < 0.001, 95% CI [−0.79, −0.29], *R* ^*2*^ = 0.33; no longer significant after controlling for *responsibility* in **path c’** *b* = −0.24, *SE* = 0.12, *p* = 0.05, 95% CI [−0.49, −0.01]), as compared to *non‐science* graduates (total effect in **path c** *b* = −0.60, *SE* = 0.11, *p* < 0.001, 95% CI [−0.86, −0.39], *R* ^*2*^ = 0.29; smaller drop in significance after controlling for *responsibility* in **path c’** *b* = −0.28, *SE* = 0.12, *p* = 0.01, 95% CI [−0.53, −0.07]).

Finally, we ran the same models with *dangerousness* as the mediator (Figure 6c) and found no significant overall mediation, as the indirect effect coefficient of *case* on *sentencing* through assumed offender *dangerousness* was not significant (path a*b *b* = 0.06, *SE* = 0.03, *p* = 0.02, 95% CI [−0.01, −0.11]) and the relationship between *case* and *sentence* remained significant after controlling for *dangerousness* (**path c’** *b* = −0.58, *SE* = 0.09, *p* < 0.001, 95% CI [−0.76, −0.42]). Similar effects in each path and non‐significant mediations persisted when we ran this model separately for people with a *science* versus *non‐science education*, although *dangerousness* had a partial effect on sentencing for *non‐science* graduates (Appendix D: Table D1).

## 4 DISCUSSION

This study examined the sentencing preferences of people with a formal education in science as compared to those that identified as non‐science graduates, for criminal offenders presented either with or without a neurobiological defense. Previous research has indicated that lay participants may generally support sentencing mitigation when presented with criminological (Berryessa, 2021) or neuroscientific (Shariff et al., 2014) explanations on how “external pressures” may bear on an offender's capacity to control their behavior and make good decisions (Berryessa, 2021; Wilson, 2012). However, our findings suggest that those with formal science education might use different information in deciding this type of support for mitigation or other preferences for criminal sentencing, as compared to non‐science graduates. Most significantly, as compared to their views on the baseline case, science graduates in our sample showed a preference for less severe sentencing options when neurobiological influences were described to have affected an offender's behavior, which was mediated by the degree to which they believed the offender was less responsible and had less free will over his actions. Indeed, these results compliment previous work suggesting that more mechanistic understandings of human behavior may diminish beliefs in free will and reduce support for retribution (Shariff et al., 2014).

Additionally, the fact that these results were not observed for non‐science graduates in our sample aligns with previous research showing that lay participants often may not link free will and perceptions of guilt or responsibility in cases involving evidence of neurobiological influences to an offender's behavior (Berryessa et al., 2021). In fact, those in our sample who were non‐science graduates appeared to put higher weight on perceptions of dangerousness, rather than free will or responsibility, when considering the punishment of offenders across all three presented cases, and most strongly when neurobiological evidence was presented. This also complements previous work showing that evidence on an offender's neurobiological characteristics may lead many members of the lay public to believe that he poses an increased risk to public safety (Berryessa, 2020; Heine et al., 2016). Thus, as Carey and Paulhus (2013) argue, these results support that those with increased scientific knowledge about behavior may be more likely than those without such knowledge to draw from neurobiological information and intuitively link views on punishment to mitigated judgements about free will and responsibility.

Our results also support the notion that science education may shift people's beliefs about the causes of –and legal responses to– offending more generally and not necessarily only in cases involving neurobiological evidence (Carey & Paulhus, 2013; Shariff et al., 2014). As compared to non‐science graduates, science graduates were more supportive of less punitive sentencing options regardless of whether scientific evidence was presented and assumed lower free will levels for offenders across all presented cases, including in the baseline case in which no scientific information was present. Therefore, similar to arguments in previous literature, this study suggests that having a formal science education might lead individuals to more generally shift support away from support for retributive responses to crime (Carey & Paulhus, 2013; Katz & Fondacaro, 2021), and particularly when neurobiological explanations are expressly provided.

Further, both science and non‐science graduates in our sample were significantly less punitive towards an offender whose behavior was said to be influenced by an innate neurobiological characteristic (i.e., genetic mutation), as compared to when no neurobiological information was presented. This adds to a mixed body of existing work on the effects of genetic explanations of behavior on sentencing decisions (Berryessa, 2020; Denno, 2011; Peters et al., 2021; Xu et al., 2021), with some experimental studies suggesting that attributing criminal behavior to genetic or other innate neurobiological factors, as an offender's deep‐rooted, permanent, and untreatable characteristics, can increase support for his punishment (Berryessa, 2020, 2022; Cheung & Heine, 2015; Xu et al., 2021). The lack of interaction between education group and the provision of neurobiological explanations might suggest that both science and non‐science graduates are receptive to genetic explanations of behavior. Interestingly, however, reduced perceptions on levels free will and responsibility mediated reduced support for punishment only for those with a formal science education. Dangerousness also was not found to fully mediate punishment support among non‐science graduates. It is notable that non‐science graduates from fields such as the humanities and law may be more sensitive to societal and public safety perspectives of crime. Future research should examine other factors that could help to explain why non‐science graduates may support mitigation based on genetic evidence.

This study does have several limitations to note. The online recruitment resulted in a limited, snow‐ball sample of university graduates for this study. Snowball sampling is a common practice in online experiments, especially those targeting particular populations (Atkinson & Flint, 2001; Zhong et al., 2014). However, such a convenience sample may compromise the independence of the observations in this study and limits the generalizability of our results –which should be replicated with probability and online crowdsourced samples. Our sample frame of biobehavioral science graduates provides initial insights into the effects of having a science background on support for sentencing, but this limited sample should be expanded in future research, which would also allow for a sufficient sample‐size to additionally compare sub‐disciplines of biological and behavioral sciences. It is important to acknowledge that this study included a diverse sample of graduates from different years and with different career lengths and trajectories, thus the differences between science and non‐science graduates cannot be strictly attributed to receiving a formal education. Indeed, in our study, the science education group represents an effort to distinguish individuals with a higher education science background, but future studies could find different effects when studying changes in attitudes from before to after completing formal education, particular professional expert populations, or those holding master's or doctorate degrees in biobehavioral science.

The experimental method using contrastive vignettes allows for isolating effects of particular variables on perceptions of punishment and sentencing determinations (Auspurg & Hinz, 2014; Berryessa, 2021). However, this study used a single vignette and instance of a criminal offense without providing multiple instances representing each criminal case and neurobiological defense. Future research could expand the present findings to increase cross‐stimulus generalization and ecological validity (Evans et al., 2015) by presenting various innate and acquired disorders and measuring participants' views across many criminal cases.

A related limitation was that participants each received two vignettes: the first without a neurobiological explanation and the second including one of two neurobiological explanations for the criminal behavior. This order may have primed participants to anchor their judgments of the second case on the initial case without a neurobiological explanation (Enough & Mussweiler, 2001). However, our experiment specifically aimed to measure how punishment attitudes are shifted in light of neurobiological evidence, and therefore we chose to include a baseline without any psychological or neurobiological information. In this study, scientists and non‐scientists still had different sentencing preferences between the baseline and neurobiological cases. Moreover, despite not using psychological evidence as a control, innate versus acquired neurobiological evidence was indeed judged differently, with only genetic evidence significantly mitigating sentences. Future work should consider a fully‐crossed experimental design that uses psychological evidence in the control condition in order to account for priming effects and any effects of adding explanatory evidence of any kind.

Ultimately, this research also has potential implications for the criminal‐legal system and particularly related to the synthesis of complex biobehavioral evidence in sentencing. Scholars have argued that assessing specialized knowledge, such as neurobiological evidence in sentencing considerations, might be a type of skilled judgment that could potentially fall prey to misperceptions in the absence of sufficient skill or knowledge (Ahn et al., 2006; Kahneman, 2003, 2011; Kahneman & Klein, 2009). Indeed, both judges (Berryessa, 2018b) and the lay public (Berryessa, 2020; Racine et al., 2005, 2010) have been shown to endorse common –often erroneous– stereotypes regarding the dangers posed by innate or acquired neurobiological disorders based on what is often popularized and discussed in the media (Berryessa, 2017b, 2020; Rafter, 2008).

For example, research indicates that judges' punishment decisions might be influenced by popular beliefs that people with particular neurobiological or mental disorders are potentially more resistant to treatment and rehabilitation, and therefore, more dangerous (Berryessa, 2018b). The public also seems to be driven by certain unsubstantiated beliefs regarding the effects of different types of psychological trauma on offending (Appelbaum & Scurich, 2014; Berryessa, 2021), as well as the mitigating effects of genetic versus neuroimaging evidence (Appelbaum et al., 2015) or different types of mental illness (Barnett et al., 2007) on sentencing determinations. Thus, popular and inaccurate understandings of the relationships between criminality, biology, and behavior could potentially increase support for severe sentencing among the lay public, with the rationale of preventing reoffending and upholding public safety (Batastini et al., 2018; Berryessa, 2018b; Perlin & Gould, 1995).

However, because long‐term prison sentences are costly and have been shown as potentially ineffective in reducing recidivism (Padfield & Maruna, 2006), these types of traditional punishments may not be constructive strategies for addressing or preventing many types of criminal behavior and their underlying causes (Fondacaro & O’Toole, 2015). Particularly, lengthy incarceration has been shown to lead to harmful consequences, both in prison and upon reentry, and do little to reduce recidivism for offenders with mental illness (Fazel et al., 2016). Instead, this study indicates that science education may help to promote less traditionally punitive and perhaps more treatment‐oriented responses to crime. Indeed, Gertner et al. (2021) suggest that a deeper understanding of science and its influence on behavior may help enhance fairness and better outcomes in criminal sentencing. In line with this and other literature (Clark et al., 2014; Shariff et al., 2014), it is possible that science education may aid in more accurate and informed judgements that involve neurobiological evidence, as well as in countering scientific oversimplifications and their potential effects on legal judgements (de Vel‐Palumbo et al., 2019; Shariff et al., 2014; Xu et al., 2021).

If people with a formal scientific education do indeed make use of knowledge that can result in less support for traditional, more retributive punishments, as this study suggests, this could bear important implications for decision‐making in criminal sentencing. As increasingly more neurobiological evidence is entering the courts and deliberated in sentencing decisions (Farahany, 2016), it is important to consider whether moral responsibility might be more effectively assessed from the lens of mechanistic or scientific influences on behavior during sentencing (Peters et al., 2021; Shariff et al., 2014). European courts, such as in the Netherlands, have taken some concrete steps to consider neurobiological influences to an offender's behavior and notions of moral responsibility during criminal sentencing by employing scientists to assist judges in interpreting scientific research and evidence, in order to increase and aid the court's scientific comprehension (de Kogel & Westgeest, 2015; Schleim, 2020). Thus, it is possible that providing similar opportunities for legal decision‐makers in the U.S., to increase scientific education and comprehension, could shift views and philosophy in sentencing practices.

Finally, as members of the public become increasingly exposed to science in school and interested in focusing their formal higher education on biobehavioral science (Roser & Ortiz‐Ospina, 2016), public attitudes towards punishment and mitigation could begin to shift in response (Shariff et al., 2014). The National Center for Education Statistics reports a steady annual increase in the number of university graduates from the fields of psychology, biology, and health sciences (*NCES*, 2019). The Sentencing Reform Act of 1984 stated that the U.S. Sentencing Commission should consider popular views as “means of measuring the degree to which the sentencing, penal, and correctional practices are effective in meeting the purposes of [just] sentencing” in their development of and reconsideration of sentencing guidelines and practices (28 U.S. Sentencing Code § 991(b)(2), 2018). Particularly, under this legislation, the U.S. Sentencing Commission should not only increasingly consider attributes of cases, defendants, or types of evidence that members of the public believe should be considered as mitigating to sentencing practices and guidelines, but also the philosophical premises for why the public believes that certain practices are appropriate in different sentencing contexts (U.S. Sentencing Commission, 1995; Maxfield et al., 1996). Indeed, if sentencing authorities and decision‐makers incorporate factors that could affect public intuitions regarding punishment, such as science education, this may help increase confidence in the criminal‐legal system and its legitimacy (Roberts & de Keijser, 2014; Xu et al., 2021).

Ultimately, if popular views on sentencing continue to evolve based on scientific progress and education (Bagaric et al., 2019; Berryessa, 2021; Gertner et al., 2021), our results help to provide nascent data on the philosophical premises that may underlie support for mitigating circumstances in sentencing practices involving neurobiological explanations for offending. Specifically, both “just deserts” (i.e., sentencing should be proportionate to an offender's moral responsibility) and utilitarianism (i.e., in relation to public safety and safeguarding communities) are explicitly reflected as philosophical premises of the U.S. sentencing guidelines (U.S. Sentencing Commission, 1995; Rappaport, 2003). This study draws a link between higher scientific education and reduced punitiveness as mediated by factors that speak to an offender's moral responsibility but not his dangerousness. This finding potentially suggests that people with a science education may be more likely to focus more on notions of “just deserts,” as compared to utilitarian motives, in punishment when considering whether an offender's sentence should be mitigated. Of course, more research is required to gain a comprehensive picture of how science‐informed perspectives could shape attitudes towards punishment and the evolution of sentencing standards based on scientific evidence. Yet, as the Commission should consider the public's views on the philosophical grounds for mitigation and appropriate practices in different sentencing contexts (U.S. Sentencing Commission, 1995; Maxfield et al., 1996), the present work suggests that such views may begin to shift as the public becomes increasingly interested in and exposed to science.

## CONFLICT OF INTEREST

The authors have no conflict to declare.

## APPENDIX B

### Main ordinal logistic regression model validations.

In Figure B1, the logistic relationships in the model are illustrated. We also made probability predictions based on the log regression model. Multiclass confusion matrices were computed (Table B1) and the misclassification error rates were calculated. The error rate was similar in the training (45%) and testing datasets (48%). The computed multiclass confusion matrix for the training dataset shows that the model accurately predicted 59 *treatment* sentences. Inaccurate predictions fell within the nearest categories in severity, with most erroneous predictions actually being *non‐custodial* sentences (17), instead of *treatment* sentences. This model also accurately predicted 83 *1–5 years* sentences, with most errors actually being *max 1‐year* sentences (37). The model performed comparably during testing. Since the training and testing datasets included randomly selected portions of the data, the model was unable to predict within certain response categories with low response rates. The stability in predictions within and between matrices indicates a consistency and reliability in the performance of the ordinal logistic models.

**TABLE B1:** Multiclass confusion matrix describing the performance of the ordinal logistic regression model in the training and testing datasets

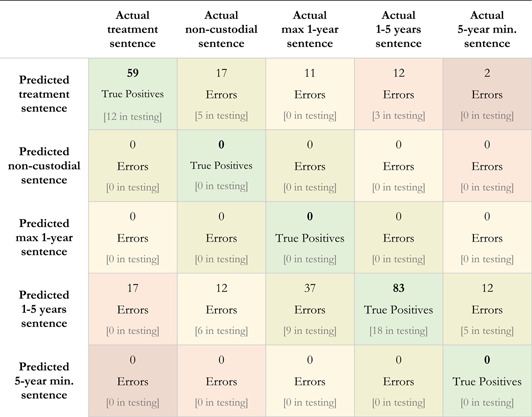

*Note*: The numbers in black font represent true positive and error rates when the model was tested on a training set, versus the predictions made on a testing subset in grey font. The color coding indicates the distance from the true positive category, with green highlighting completely correct and red highlighting completely incorrect predictions, while in between the green‐yellow predictions were close to the true values. This confusion matrix illustrates that any erroneous predictions are still centered around the true sentences given, and predictions are generally consistent across models.

## APPENDIX C

**TABLE C1:** Lists of countries of residence, nationalities, and criminal histories in each education group

	Non‐science education	Science education
Country of residence		
Argentina	0	1
Australia	1	4
Austria	0	1
Barbados	1	0
Belgium	5	3
Canada	1	1
Colombia	0	1
Croatia	0	1
Ecuador	0	1
Egypt	1	0
France	2	0
Germany	1	2
Greece	4	4
Hungary	0	1
Indonesia	1	1
Ireland	0	1
Italy	1	0
Kenya	0	1
Latvia	0	1
Malaysia	0	1
Mexico	0	1
Netherlands	19	43
New Zealand	1	1
Poland	1	0
Portugal	2	0
Romania	1	0
Saint Kitts and Nevis	1	0
South Africa	0	2
Spain	4	3
Sweden	1	1
Switzerland	0	1
United Arab Emirates	0	2
United Kingdom	3	10
United States	4	16
Nationality		
Argentina	1	0
Belgium	7	5
Croatia	0	1
Estonia	0	1
France	1	1
Germany	0	3
Greece	0	1
India	0	2
Indonesia	0	1
Iraq	0	1
Lebanon	1	0
Malaysia	0	1
Morocco	0	1
Mozambique	0	1
Netherlands	40	66
Russian Federation	1	1
Saudi Arabia	0	1
Spain	1	0
United Kingdom	3	4
United States	0	14
Criminal History		
Arrest but no conviction	0	1
Close family member imprisoned	2	6
Convicted of crime and served time in jail/prison	1	0
Convicted of crime but served no jail time	1	0
I haven't had any significant encounters with the law	32	53
Traffic violation	19	45

## APPENDIX D

### All computed mediation paths

**TABLE D1:** Mediation results with Case as the predictor and Sentence as the outcome variable in all computed models

Mediation model	*b*	Std. Error	Sig.	95% CI
A. Mediator: *Offender Free Will*				
1. All participants				
Path a	−17.50	1.65	<0.001	−20.72, −14.32
Path b	0.028	0.004	<0.001	0.02, 0.03
Path c	−0.61	0.09	<0.001	−0.76, −0.41
Path a*b	−0.49	0.09	**<0.001**	−0.57, −0.24
Path c’	−0.12	0.09	0.21	−0.37, 0.007
2. *Science education*				
Path a	−17.90	2.51	<0.001	−23.12, −13.08
Path b	0.024	0.004	<0.001	0.02, 0.03
Path c	−0.54	0.13	<0.001	−0.80, −0.30
Path a*b	−0.42	0.11	**<0.001**	−0.67, −0.26
Path c’	−0.12	0.14	0.41	−0.39, 0.16
3. *Non‐science education*				
Path a	−17.10	2.05	<0.001	−20.9, −13.1
Path b	0.025	0.02	0.09	0.007, 0.03
Path c	−0.65	0.21	0.001	−0.89, −0.39
Path a*b	−0.43	0.26	0.10	−0.57, 0.11
Path c’	−0.22	0.16	0.16	−0.58, −0.02
B. Mediator: *Offender Responsibility*				
4. All participants				
Path a	−16.30	1.67,	<0.001	−19.48, −12.56
Path b	0.019	0.002	<0.001	0.01, 0.02
Path c	−0.56	0.09	<0.001	−0.74, −0.39
Path a*b	−0.31	0.05	**<0.001**	−0.42, −0.22
Path c’	−0.25	0.09	0.004	−0.42, −0.08
5. *Science education*				
Path a	−14.40	2.61	<0.001	−19.86, −9.43
Path b	0.019	0.003	<0.001	0.01, 0.03
Path c	−0.52	0.12	<0.001	−0.79, −0.29
Path a*b	−0.28	0.07	**<0.001**	−0.44, −0.16
Path c’	−0.24	0.12	0.05	−0.49, −0.01
6. *Non‐science education*				
Path a	−18.07	2.08	<0.001	−22.16, −14.01
Path b	0.018	0.004	<0.001	0.01, 0.03
Path c	−0.60	0.11	<0.001	−0.86, −0.39
Path a*b	−0.32	0.08	**<0.001**	−0.48, −0.17
Path c’	−0.28	0.12	0.01	−0.53, −0.07
C. Mediator: *Offender Dangerousness*				
7. All participants				
Path a	3.68	1.31	0.01	1.09, −6.21
Path b	0.015	0.003	<0.001	0.01, 0.02
Path c	−0.52	0.08	<0.001	−0.69, −0.37
Path a*b	0.06	0.03	0.02	−0.01, −0.11
Path c’	−0.58	0.09	<0.001	−0.76, −0.42
8. *Science education*				
Path a	3.04	1.99	0.13	−0.77, 6.89
Path b	0.011	0.01	0.02	0.002, 0.02
Path c	−0.47	0.11	<0.001	−0.71, −0.27
Path a*b	0.03	0.06	0.02	−0.01, −0.11
Path c’	−0.50	0.12	<0.001	−0.75, −0.29
9. *Non‐science education*				
Path a	4.31	1.72	0.01	1.01, 7.75
Path b	0.017	0.01	<0.001	0.008, 0.03
Path c	−0.59	0.11	<0.001	−0.82, −0.39
Path a*b	0.03	0.06	**0.01**	−0.01, −0.09
Path c’	−0.66	0.12	<0.001	−0.91, −0.47

*Note*: **Path c** represents the total effect of *case* on *sentencing* for all three criminal cases, while path c’ represents the mediated relationship and tests whether **path c** is weaker or no longer significant after controlling for the influence of the mediator variable. **Path a*b** presents the indirect effect coefficient of *case* on *sentencing* through the mediator variable. Statistically significant mediated paths a*b are shown in bold.
